# Dengue Type Four Viruses with E-Glu_345_Lys Adaptive Mutation from MRC-5 Cells Induce Low Viremia but Elicit Potent Neutralizing Antibodies in Rhesus Monkeys

**DOI:** 10.1371/journal.pone.0100130

**Published:** 2014-06-24

**Authors:** Hsiao-Han Lin, Hsiang-Chi Lee, Xiao-Feng Li, Meng-Ju Tsai, Hung-Ju Hsiao, Jia-Guan Peng, Shih-Che Sue, Cheng-Feng Qin, Suh-Chin Wu

**Affiliations:** 1 Institute of Biotechnology, Department of Life Science, National Tsing Hua University, Hsinchu, Taiwan; 2 Department of Virology, State Key Laboratory of Pathogen and Biosecurity, Beijing Institute of Microbiology and Epidemiology, Beijing, China; 3 Institute of Bioinformatics and Structural Biology, Department of Life Science, National Tsing Hua University, Hsinchu, Taiwan; 4 Department of Medical Science, National Tsing Hua University, Hsinchu, Taiwan; University of Rochester, United States of America

## Abstract

Knowledge of virulence and immunogenicity is important for development of live-attenuated dengue vaccines. We previously reported that an infectious clone-derived dengue type 4 virus (DENV-4) passaged in MRC-5 cells acquired a Glu_345_Lys (E-E_345_K) substitution in the E protein domain III (E-DIII). The same cloned DENV-4 was found to yield a single E-Glu_327_Gly (E-E_327_G) mutation after passage in FRhL cells and cause the loss of immunogenicity in rhesus monkeys. Here, we used site-directed mutagenesis to generate the E-E_345_K and E-E_327_G mutants from DENV-4 and DENV-4Δ30 infectious clones and propagated in Vero or MRC-5 cells. The E-E_345_K mutations were consistently presented in viruses recovered from MRC-5 cells, but not Vero cells. Recombinant E-DIII proteins of E_345_K and E_327_G increased heparin binding correlated with the reduced infectivity by heparin treatment in cell cultures. Different from the E-E_327_G mutant viruses to lose the immunogencity in rhesus monkeys, the E-E_345_K mutant viruses were able to induce neutralizing antibodies in rhesus monkeys with an almost a 10-fold lower level of viremia as compared to the wild type virus. Monkeys immunized with the E-E_345_K mutant virus were completely protected with no detectable viremia after live virus challenges with the wild type DENV-4. These results suggest that the E-E_345_K mutant virus propagated in MRC-5 cells may have potential for the use in live-attenuated DENV vaccine development.

## Introduction

Dengue virus (DENV) is a vector-borne virus that is transmitted to humans by *Aedes aegypti* and *Aedes albopictus* mosquitoes in tropical and sub-tropical areas. Disease severity ranges from asymptomatic infections to a febrile fever, or potentially life-threatening dengue hemorrhagic fever (DHF) and dengue shock syndrome (DSS). According to the World Health Organization (WHO), two-fifths of the world's population is at risk of DENV infection. 50–100 million cases occur each year, resulting in hundreds of thousands of incidents of DHF and DSS [1]. Although there are no licensed DENV vaccines to date, a tetravalent DENV-yellow fever 17D chimeric vaccine (referred to as CYD chimeras) [2] is currently being evaluated in Phase 3 trials. Other two vaccine candidates in clinical trials are based on the similar approach to construct four chimeric DENV by using either the DENV-2 PDK-53 backbone (referred to as DENVax chimeras) [3], or the DENV-4 infectious clone (strain 814669) with a 30 nucleotide-deletion in 3′ noncoding region (NCR) (referred to as DENVΔ30 chimeras) [4].

Passaging of DENVs or their derived chimeras in Vero cells has been shown to generate mutations that are specific for host cell adaptation, virus attenuation, or other properties yet to be characterized [5], [6]. Spot sequencing and TaqMan mismatch amplification mutation methods have been used to identify loss of the attenuating markers in chimeric DENV-2 PDK-53 vaccines following initial passages in Vero cells [3], [7]. Several mutations in prM-E and NS4B regions were also detected in the seed stocks of ChimeriVax-DENV vaccine development [8]. We previously found that DENV-4 infectious clone viruses propagated in MRC-5 (human fetal lung fibroblast) cells maintained greater genetic stability compared to viruses propagated in Vero cells, and a single E-Gly_345_Lys (E-E_345_K) mutation was detected in 50% of DENV-4 virus propagated in MRC-5 cells [9]. More severe DENV-induced hemorrhaging in mice was also observed following DENV-4 and DENV-4Δ30 passages in Vero cells compared to passages in MRC-5 cells [10]. It was also reported that three passages of the same backbone–cloned DENV-4 in fetal rhesus lung (FRhL) cells yielded a single E-Glu_327_Gly (E-E_327_G) mutation with enhanced heparin bindings, also resulting in a loss of infectivity and immunogenicity in rhesus monkeys [11]. Whether the increased heparin bindings of these adaptive mutations from MRC-5, Vero and FRhL cells cause the loss of immunogenicity in rhesus monkeys is still unknown.

In this study, we conducted site-directed mutagenesis on the infectious clones of DENV-4 and DENV-4Δ30 [12]. The E-E_345_K and E-E_327_G mutant viruses were obtained from DENV-4 and DENV-4Δ30 infectious clones and passaged in Vero and MRC-5 cells, respectively. The genetic stability and replication kinetics in Vero and MRC-5 cells were characterized. Enhanced heparin bindings and reduced mouse neurovirulence were observed for both E-E_345_K and E-E_327_G mutants compared to DENV-4 wild type. Since DENV-4 E-E_327_G mutant failed to induce viremia and neutralizing antibodies in monkeys [11], the present studies demonstrated that DENV-4 E-E_345_K mutant induced potent neutralizing antibodies with low viremia in rhesus monkeys and protective immunity after live virus challenges. Our findings may have important implications regarding the development of live-attenuated DENV vaccines.

## Materials and Methods

### Ethics Statement

The mouse studies were conducted in accordance with guidelines established by the Laboratory Animal Center of National Tsing Hua University (NTHU). Animal use protocols were reviewed and approved by the NTHU Institutional Animal Care and Use Committee (approval no. 09931). Mice survived from experimental studies were sacrificed using carbon dioxide (CO_2_) following ISCIII IACUC guidelines to ameliorate suffering. All experimental procedures involving monkeys were approved by the Animal Experiment Committee of Beijing Institute of Microbiology and Epidemiology, China. The animals were individually housed in cages and the room was cleaned and disinfected every 10 days. Fresh fruits and vegetables were provided twice a week. All procedures were performed under sodium pentobarbital anesthesia by trained technicians from the animal center and all efforts were made to ameliorate the welfare and to minimize animal suffering in accordance with the “Weatherall report for the use of non-human primates” recommendations.

### Cells, media, and viruses

Vero, Vero E6, and MRC-5 cells were obtained from the Bioresource Collection and Research Center of the Food Industrial Research and Development Institute, Hsinchu, Taiwan. Cells were grown in Minimum Essential Media (MEM) (Invitrogen) supplemented with 10% heat-inactivated fetal bovine serum (FBS) and 100 U/ml of penicillin G sodium-streptomycin (Invitrogen). Stock viruses were prepared from the supernatants of infected C6/36 cells grown in Hank's MEM medium (Gibco-BRL) plus 10% FBS for 6 days at 28°C. To prepare high DENV titers, virus supernatant was concentrated with a Centriplus device (10-kDa cutoff) (Amicon, Millipore) and centrifuged at 2,800 rpm for 30 min before each assay. All virus stocks were stored at −80°C until used for analysis. Virus titers were measured using 10-fold serial dilutions of culture supernatant in duplicate infections of Vero-E6 cell monolayers in 6-well plates. After incubation at 37°C for 1 h, 4 ml of medium containing 1×MEM, 1.1% methylcellulose, and 100 U/ml of penicillin G sodium-streptomycin was added to each well (4 ml/well). After 6 days post-incubation, virus plaques were stained with 1% crystal violet dye and Infectivity titers were measured in plaque forming units per ml (PFU/ml). To improve the visualization of E-E_345_K mutant viruses which produce small plaques, the infectivity titers were determined using focus-forming-unit (FFU) assay. For FFU assay, the infected cells at 6 days post-incubation were fixed in 4% formaldehyde, permeabilized with 0.05% Tween 20, reacted with HB-114 monoclonal antibody, anti-mouse HRP secondary antibody, and stained with Liquid DAB-Plus Substrate Kit (Invitrogen).

### Construction and preparation of wild type and mutant viruses from DENV-4 and DENV-4Δ30 Infectious clones

The infectious clones of DENV-4 and its 3′ NCR deletion mutant DENV-4Δ30 contained the full-length DENV cDNA corresponding to the vaccine candidate strain 814669 [12], [20], [21], [22], [23]. Site-directed mutagenesis was performed with overlapping PCR to generate each mutant cDNA clones of DENV-4 E-E_345_K, DENV-4 E-E_327_G, and DENV-4Δ30 E-E_345_K. To construct DENV-4 E-E_345_K we used the primer pairs *NsiI*-f and E_345_K-r and E_345_K-f and *StuI*-r. In the second pair, E_345_K-f changed the E gene amino acid no. 345 from Glu to Lys (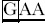 to 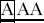). To construct DENV-4 E_327_G we used the primer pairs *NsiI*-f and E_327_G-r; and E_327_G-f and *StuI*-r. In the second pair, E_327_G-f changed the E gene amino acid no. 327 from Glu to Gly (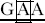 to 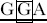). Infectious cDNA clone of the parental virus DENV-4 was used as template for PCR reactions. The second round was executed using the same primer pair *NsiI*-f and *StuI*-r. We cloned the *NsiI*-*StuI* PCR fragments (2,003-bp) into a pJET1.2/blunt cloning vector (Fermentas Life Sciences) for amplification. To introduce changes at E protein residues 345 and 327, a 2 kb region flanked by *NsiI* and *StuI* restriction enzyme sites in the DENV-4 or DENV-4Δ30 infectious clone was replaced with *NsiI*-*StuI* fragments derived from confirmed clones containing E-E_345_K or E-E_327_G mutations. All the nucleotide sequences were confirmed by Mission Biotech Inc., Taipei, Taiwan.

Infectious clone plasmids were linearized by cleavage with the *Kpn*I restriction enzyme, added to a transcription reaction mixture, and transcribed using SP6 RNA polymerase and the RiboMAX large-scale RNA production system (Promega). Full-length RNA transcripts were further capped with m^7^G(5′)ppp(5′)G at the RNA 5′ end using a ScriptCap Capping enzyme (EPICENTRE). After incubation at 37°C for 1 h, RNA product was purified with TRIzol LS reagent (Invitrogen) according to the manufacturer's instructions. Prior to RNA transfection, subconfluent Vero and MRC-5 cells (in 6-well plates) were rinsed with serum-free medium. The transfection mixture was prepared by adding 4 µl of DMRIE-C reagent (Invitrogen) to 1 ml of serum-free medium, followed by mixing with 10 µg of the RNA product. The transfection mixture was added directly to cell monolayers. After incubation for 18 h at 37°C, DMEM +10% FBS was added to each well. Culture supernatants were collected at 8 days post-transfection. All virus stocks were stored at −80°C until used.

### Sequencing of DENV-4 and DENV-4Δ30 wild type and mutant viruses

Viral RNAs were extracted from culture supernatants using TRIzol reagent (Invitrogen), and the cDNA synthesized by reverse transcription using Superscript II RTase (Invitrogen). The double-stranded DNA was then generated by PCR using Platinum *Pfx*DNA polymerase (Invitrogen). Four overlapping fragments that span the entire DENV-4 genome were produced using 4 sets of forward and reverse primers: (i) W01/W02R, (ii) W03/W04R, (iii) W05/W06R, and (iii) W07/W08R reported previously(9). The nucleotide sequences of each of the four fragments were determined by Mission Biotech Inc., Taipei, Taiwan. The cloned DNA segments that span the entire DENV-4 genome were mapped using the software Lasergene version 6.00 (DNASTAR, Inc. USA) to determine the extent of overlapping sequences.

### Infectivity of wild type, DENV-4 E-E_345_K and E-E_327_G mutant viruses by GAGs

100 PFU of DENV-4 or clone-derived DENV-4 E-E_345_K and E-E_327_G mutants were incubated with the concentrations of 10 ug/ml of heparin (Sigma), heparan sulfate (Sigma), chondroitin 4-sulfate (chondroitin sulfate A sodium salt) (Sigma), β-Heparin (dermatan sulfate sodium salt, chondroitin sulfate B sodium salt) (Sigma), chondroitin 6-sulfate (chondroitin sulfate C sodium salt) (Sigma), hyaluronic acid sodium salt (Sigma), heparin disaccharide IV-A, I-H, III-S or I-S sodium salt (Sigma) for 1 hour at 37°C in a CO2 incubator. Confluent monolayers of Vero-E6 cells in 6-well plates were rinsed with PBS and incubated with the mixture at 37°C for 1 hour. Next, 4 ml of medium containing 1× EMEM, 1.1% methylcellulose, and 100 U/ml of penicillin G sodium-streptomycin were added to each well. Virus titers were determined by plaque or focus-forming assays of Vero-E6 cells. The relative infectivity was determined as the percentage of GAGs-treated infectious titers to protein A sepharose bead –treated infectious titers.

### Infectivity inhibition assays using Heparin-Sepharose and Hyaluronic Acid-EAH Sepharose beads

50 µM heparin-Sepharose, hyaluronic acid-EAH Sepharose and and Control protein A-Sepharose beads (Pharmacia, Uppsala, Sweden) were respectively suspended in phosphate-buffered saline (PBS) and equilibrated before use by pelleting and washing three times in Hank's balanced salt solution (Invitrogen) containing 0.2% bovine serum albumin (Gibco) (HBSS-BSA) plus 10 mM HEPES (pH 8.0). Next, 10^5^ FFU of DENV-4 wild type, E-E_345_K and E-E_327_G mutants was diluted in the tube before mixing with or without Sepharose beads at 4°C for 6 hr. All viruses were diluted in HBSS-BSA plus with or without Sepharose beads. Virus-bead mixtures were centrifuged for 5 min at 6,000×*g* at 4 °C to pellet the Sepharose beads. Infectious titers in supernatants were determined by plaque or focus-forming assays of Vero-E6 cells. The relative infectivity was determined as the percentage of beads-treated infectious titers to beads-untreated infectious titers.

### Recombinant DIII protein-heparin binding ELISA assay

The envelope protein domain III (DIII) genes of wild type, E-E_345_K and E-E_327_G viruses were cloned into pET22b (+) expression vector (Novagen) with an additional C-terminal 6x histidine gene to facilitate purification. The vectors were transformed into E.coli BL21 (DE3) cells (Invitrogen) and stimulated with 1 mM IPTG. DIII proteins obtained from inclusion bodies were purified using nickel-chelated resin (TOSOH) affinity chromatography under denaturing condition after cells were homogenized at 15K psi and re-suspended in 8M urea. Purified DIII proteins were eluted on 30–40% buffer B (300 mM Tris, 50 mM NaCl, 500 mM imidazole, 5% glycerol, at pH 7.4), concentrated by 30,000 centrifugal filters (MILLPORE), and analyzed by SDS-PAGE gel stained with Coomassie blue. For heparin binding ELISA assay, 5 mg heparin was added to 5 mg N-ethoxycarbonyl-2-ethoxy-1,2-dihydroquinoline (Sigma) in 500 µl of ethanol and incubated at room temperature for 1 h, then 10 mg of BSA was added and incubated at 4°C overnight. Unconjugated heparin was removed by centrifugation at 3000 g using a spin concentrator (50-kDa cutoff) (Amicon, Millipore). ELISA plates were coated with 50 µl BSA–heparin (5 µg per ml) per well in a carbonate buffer (29 mM Na_2_CO_3_, 71 mM NaHCO_3_) pH 9.6 at 4°C overnight. Plates were blocked with 200 µl blocking buffers (PBS with 1% BSA) for 1 h and then recombinant DIII proteins added for 1 h at room temperature. Plates were washed with PBST (PBS with 0.05% Tween-20) and incubated with mouse anti-His antibody for 1 h at 37°C. Plates were washed with PBST and then probed with anti-mouse–HRP conjugate antibody. Plates were again washed and TMB substrate buffers (BioLegend) was added for 15 min. Color reaction was arrested with 2N H_2_SO_4_ and plates were read at 450 nm. reader.

### Mouse neurovirulence

All mice were housed at the National Tsing Hua University barrier facility. All experiments were conducted in accordance with guidelines established by the Laboratory Animal Center of National Tsing Hua University (NTHU). Animal use protocols were reviewed and approved by the NTHU Institutional Animal Care and Use Committee (permit number: 09734). Inoculum were prepared by diluting virus stocks in HBSS containing 0.4% bovine serum albumin fraction V (Gibco) (HBSS-0.4% BSA fraction V) immediately prior to inoculation. For measuring neurovirulence, litters of newborn (<24 h) outbred white ICR mice (BioLASCO Taiwan Co., Ltd) were inoculated intracranially with 30 µl of Mock diluent or diluent containing 10^4^ FFU of the wild type and mutant DENV-4 and DENV-4Δ30 as described previously [3], [24], [25]. Each treatment in this experiment was over 12 mice and in at least two litters. HBSS-0.4% BSA fraction V was used as a diluent. Each group consisted of at least 10 newborn mice per treatment. Mice were observed for 18 days.

### Infectivity and neutralizing antibody responses in rhesus monkeys

Six monkeys, weighing 3.6 to 4.8 kg, were prescreened for negativity for antibodies against DENV by IFA. Animals were randomly divided into two groups and immunized subcutaneously (s.c.) in the deltoid region with 0.5 ml of cloned DENV-4 or DENV-4 E-E_345_K mutant containing 10^5^ FFU. Blood was collected daily for 10 days to detect viremia. Blood samples for neutralizing-antibody tests were taken before immunization and then on days 0, 14 and 28 post immunization. On day 157 post immunization, the E-E_345_K mutant immunized monkeys were challenged by s.c. inoculation of 0.5 ml containing 10^5^ PFU of DENV-4 wild type. Blood was collected daily for 10 days to detect viremia after live virus challenges. Blood samples for neutralizing-antibody were measured by neutralization assay on days 15 and 30 post-challenge. The levels of viremia in immunized monkeys were measured by quantitative real-time reverse transcriptase PCR. Briefly, viral genomic RNA was extracted from 200 µl of serum or an equal volume of sample serum by using the PureLink RNA minikit (Invitrogen, USA) according to the manufacturer's instructions. RNA was eluted in 50 µl of RNase-free water, aliquoted, and stored at −80°C until use. The target region for the assays was based on the C protein region of the genome of DENV-4. The number of PFU in serum detected was calculated by generating a standard curve from 10-fold dilutions of RNA isolated from a serum sample containing a known number of PFU, the titer of which was determined by plaque assay. Neutralizing antibody titers in sera were measured using a serum dilution-plaque reduction neutralization test (PRNT). We incubated 100 PFUs of the wild type DENV-4 with equal volumes of serial two-fold dilutions of heat-inactivated serum at 37°C for 1 h. Six-well plates of Vero-E6 cells were inoculated with the serum-virus mixture and incubated at 37°C 1 h. Plates were treated as described for the plaque titration protocol. Neutralizing antibody titers were identified as the highest serum dilution reducing the number of virus plaques by 50%. The 50% neutralization inhibition dose (PRNT_50_, the geometric reciprocal of serum dilution yielding 50% reduction in virus titer) was obtained using using GraphPad Prism version 5.01 (GraphPad Software, Inc.)

### Surface mapping of electrostatic fields

Molecular modeling of E-E_345_K and E-E_327_G mutations (SWISS-MODEL, http://swissmodel.expasy.org) was based on the DENV-4 E-DIII structural model as determined by nuclear magnetic resonance (NMR) spectroscopy (Protein Data Bank code 2H0P). Surface mapping of the DENV-4 E-DIII electrostatic field was displayed using PyMOL software (v 0.99, Delano Scientific). Blue and red colors in Figure S2 denote positive and negative charges, respectively.

### Statistical analysis

Statistical analyses were carried using GraphPad Prism (GraphPad Software, Inc.). Statistical significance of differences between groups was assessed using two-tailed Student's *t* test. Differences with a *P* value of less than 0.05 (*) were considered statistically significant.

## Results

### Generation of E-E_345_K mutant viruses from DENV-4 and DENV-4Δ30 infectious clones

We conducted site-directed mutagenesis of these mutations on the two infectious cDNA clones, DENV-4 and DENV-4Δ30, to generate E-E_345_K mutant viruses. RNA transcripts were obtained by incubating the cDNAs with SP6 RNA polymerase and rNTPs for 2 h, and then capping the transcribed RNA *in vitro* with GTP and a 5′-Cap capping enzyme for 1 h at 37°C. The *in vitro* RNA transcripts were analyzed by RNA gel electrophoresis to confirm high quality (data not shown). RNA transcripts were transfected into Vero or MRC-5 cells (i.e. P0) and propagated via four consecutive passages (i.e. P1, P2, P3, P4) at MOI  = 0.01 to avoid the influence by accumulation of defective interfering particles and enhance the possibility for cell adaptation. Small plaque formation with reduced cytopathogenicity was observed in Vero-E6 cells for E-E_345_K mutants derived from both DENV-4 and DENV-4Δ30 infectious clones.

**Figure 1:**
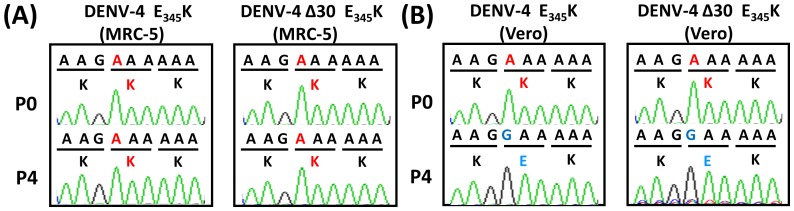
Electropherograms from sequence analyses of mutant virus derived from DENV-4 and DENV-4Δ30 clones passaged in Vero and MRC-5 cells. (A) DENV-4 and DENV-4Δ30 E-E_345_K in MRC-5 cells, (B) DENV-4 and DENV-4Δ30 E-E_345_K in Vero cells, all passaged four times each in Vero and MRC-5 cells.

Virus stocks obtained from P4 were extracted and their sequences were analyzed using RT-PCR and the results were compared to the sequences from the *in vitro* RNA transcripts (P0) to rule out the in vitro transcription errors. Results indicate that the E-E_345_K mutations of the DENV-4 and DENV-4Δ30 clones were consistent during all passages in MRC-5 cells (Fig. 1A) but were reverted to the wide type sequences in P4 Vero cells (Fig. 1B). In contrast, the consensus wild-type sequence of DENV-4 and DENV-4Δ30 was stable in P0 and P4 Vero and MRC-5 cells. Therefore, we harvested E-E_345_K mutant stocks from MRC-5 cells and the wild-type stocks from Vero cells for the following experimental investigation.

### Replication kinetics of E-E_345_K mutants in Vero and MRC-5 cells

**Figure 2:**
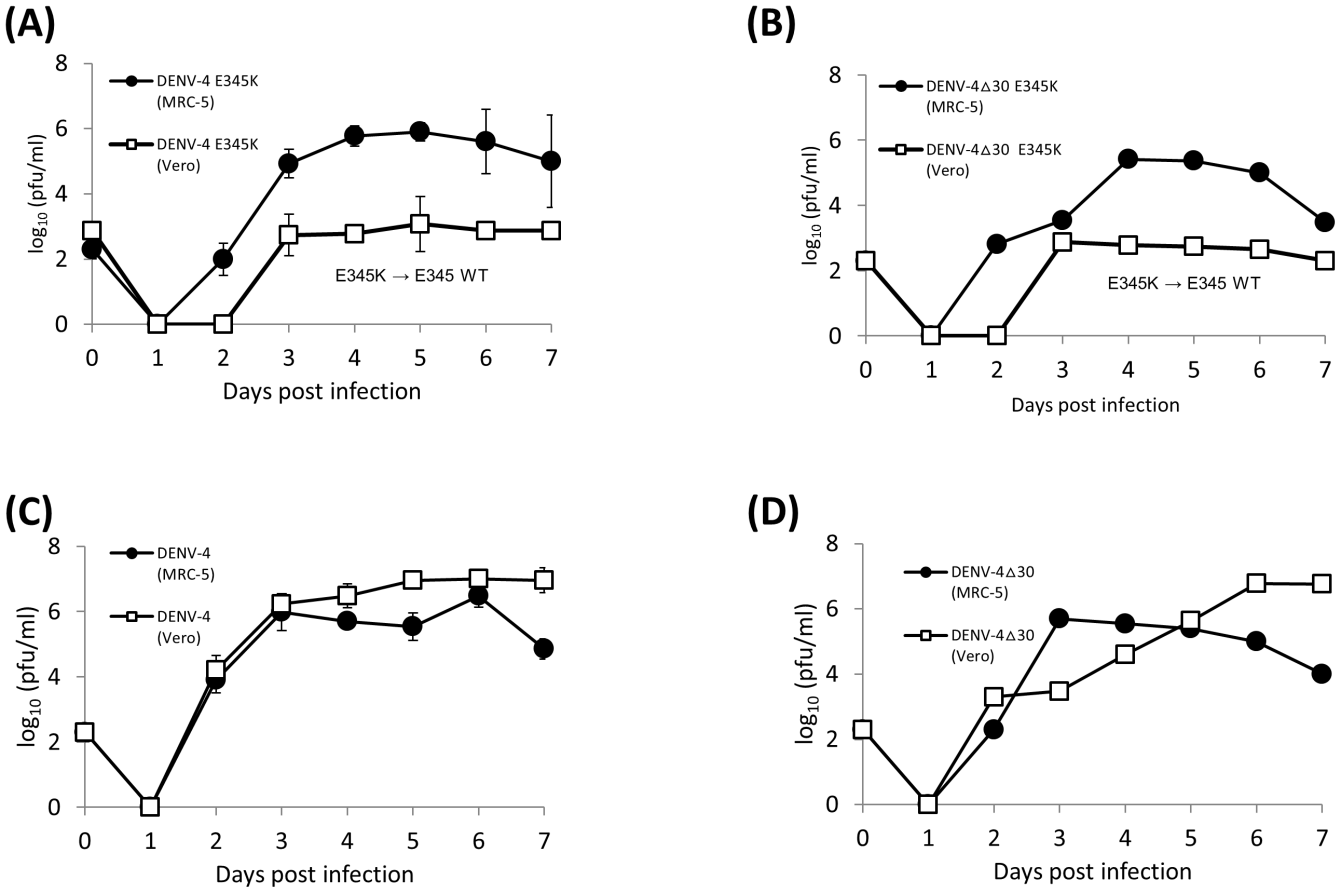
Virus replication kinetics in Vero(□) and MRC-5(•) cells infected at MOI  = 0.01 by (A) DENV-4 E-E_345_K, (B) DENV-4Δ30 E-E_345_K, (C) DENV-4 wild type, and (D) DENV-4Δ30 wild type virus. Bars represent means with standard deviation from two independent sets of experiments.

We measured the replication kinetics of E-E_345_K mutants in MRC-5 and Vero cells, respectively. The E-E_345_K mutant derived from DENV-4 infectious clone grew productively in MRC-5 cells (FIG. 2A). In contrast, the E-E_345_K mutant grew poorly in Vero cells (approximately 2-3 log lower titers). However, sequencing the virus stocks from Vero cells revealed the reversion of E_345_K to E_345_ WT sequence. (FIG. 2A) Similarly, only the E-E_345_K mutant derived from DENV-4Δ30 infectious clone grew productively in MRC-5 cells but grew poorly in Vero cells with also the reversion of E_345_K to E_345_ WT sequence (FIG. 2B). Wild type DENV-4 and DENV-4Δ30 (FIG. 2C, 2D) grew productively in both Vero and MRC-5 cells. Thus, the results demonstrated the genetic stability and replication kinetics of E-E_345_K mutants were consistently presented in MRC-5 cells.

### Infectivity inhibition by heparin treatments with wild type, E-E_345_K and E-E_327_G mutant viruses

**Figure 3:**
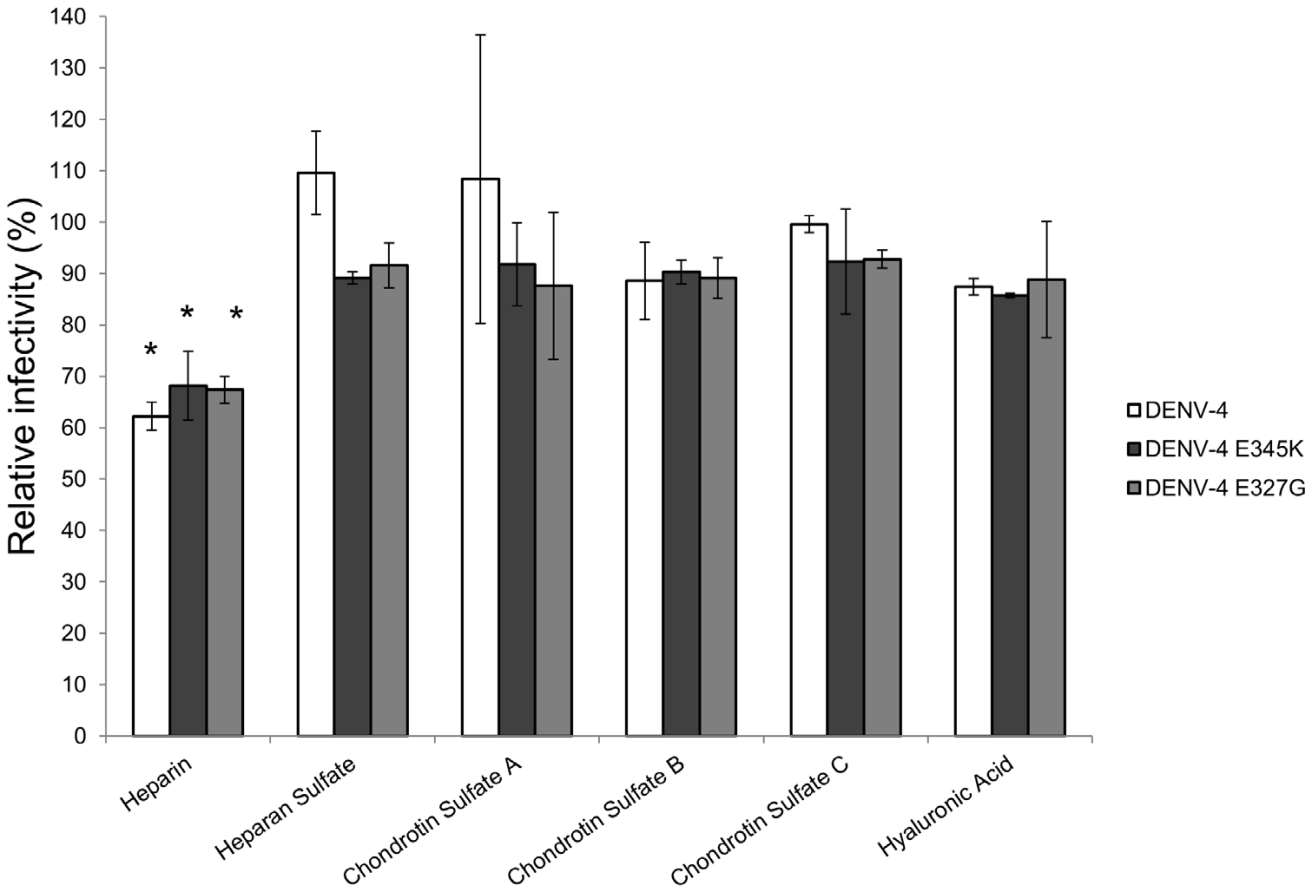
Effects of various GAGs at the concentration of 10 µg/ml on the inhibition of virus infectivity in Vero-E6 cells as determined by plaque percentages for E-E_345_K mutant viruses and E-E_327_G mutant viruses compared to their untreated controls. (* indicates statistical significance at p<0.05). Bars represent means with standard deviation from two independent sets of experiments.

As the E-E_327_G mutant virus was reported to give enhanced heparin binding [11], we also conducted site-directed mutagenesis of E-E_327_G mutation on the DENV-4 infectious cDNA clone. We harvested E-E_327_G mutant stocks from Vero cells. We then examined the infectivity inhibition by different types of soluble glucosaminoglycans (GAGs), including heparan sulfate, heparin, chondroitin sulfate A, B, C, and hyaluronic acid. 100 FFU of DENV-4 wild type, E-E_345_K or E-E_327_G mutants were pre-treated with various GAGs at the concentration of 10 µg/ml. Compared to the untreated controls (no GAGs), the FFU percentages of DENV-4 wild type, E-E_345_K and E-E_327_G mutant were reduced by the treatment with heparin, but not with heparan sulfate, hyaluronic acid, chondroitin sulfate A, B, or C (Fig. 3). Only the reduction of FFU percentages for the treatments with heparin were statistically significant (p<0.05).

### Heparin binding specificity of E-E_345_K and E-E_327_G mutations

**Figure 4:**
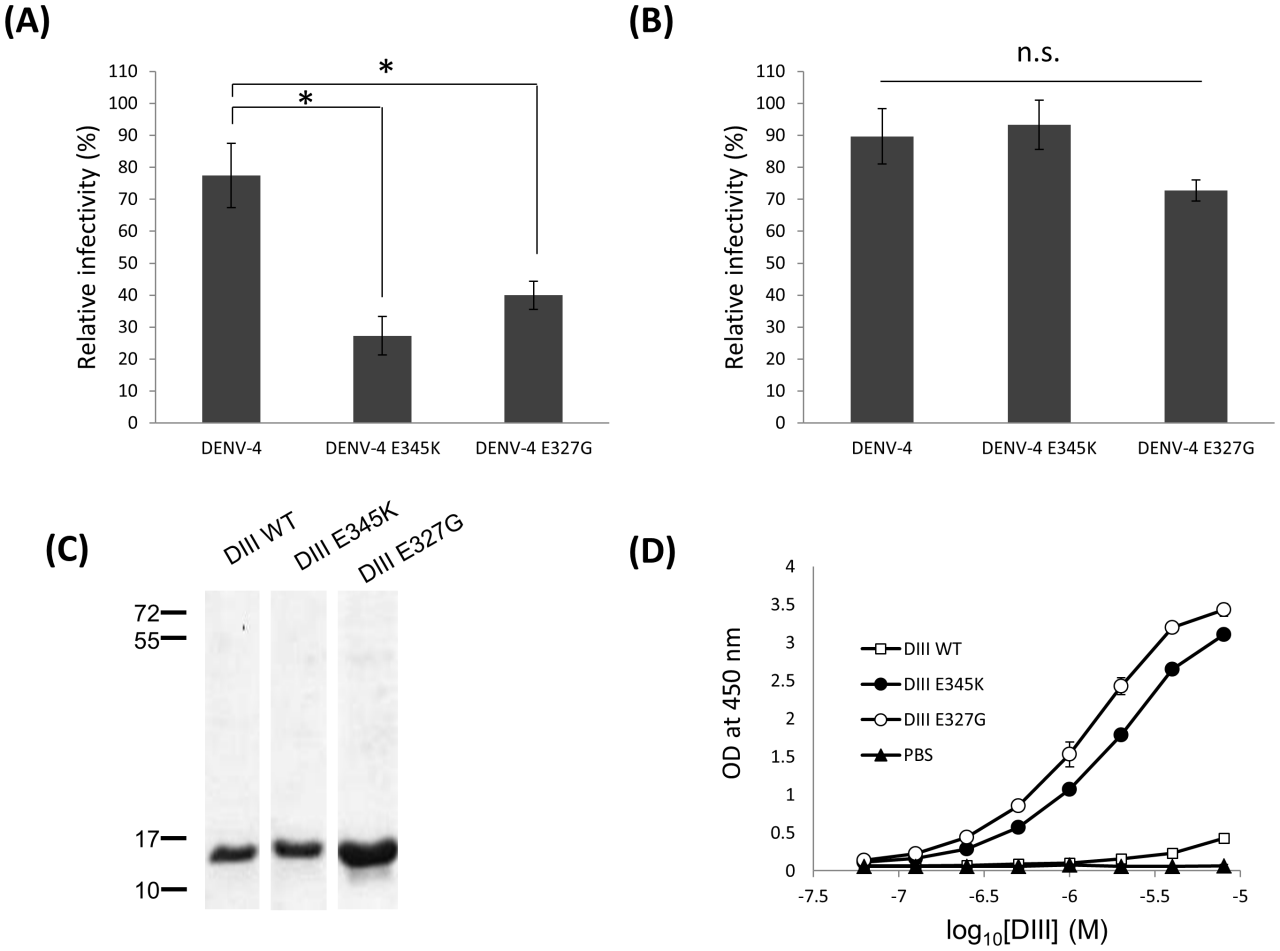
Effects of heparin binding on E-E_345_K and E-E_327_G mutants infectivity and recombinant DENV-4 E-DIII proteins. Results from heparin binding assays of DENV-4 E-E_345_K, DENV-4 E-E_327_G, and DENV-4 viruses, determined by virus removal using (A) heparin-sepharose beads and (B) hyaluronic acid-EAH sepharose 4B beads. Recombinant DENV-4 wild type and mutants E-DIII proteins were expressed from E. coli (C) and the purified proteins and stained with Coomassie blue. (D) heparin binding ELISA was used to determine E-DIII proteins-heparin binding activity. (* indicates statistical significance at *p*<0.05; n.s. indicates none statistical significance).

We further used heparin and hyaluronic acid beads to examine the specificity of infectivity inhibition. The relative FFU percentages of E-E_345_K and E-E_327_G mutants were significantly reduced by heparin binding compared to the DENV wild type (FIG. 4A). In contrast, no significant reductions of the bindings to hyaluronic acid beads were found among the wild type, E-E_345_K, and E-E_327_G mutant viruses (FIG. 4B). Moreover, recombinant E-DIII proteins of the wild type, E-E_345_K and E-E_327_G mutants were expressed from E. coli and the purified proteins were obtained as shown in FIG. 4C. Heparin binding ELISA demonstrated the dose-dependent increases in the heparin bindings by E-E_345_K and E-E_327_G E-DIII proteins but not the wild type E-DIII protein and PBS (FIG. 4D). However, no significant difference of the increased heparin binding ELISA was found between the E-E_345_K and E-E_327_G E-DIII proteins.

### Mouse neurovirulence of E-E_345_K mutant viruses from DENV-4 and DENV-4Δ30 infectious clones

**Figure 5:**
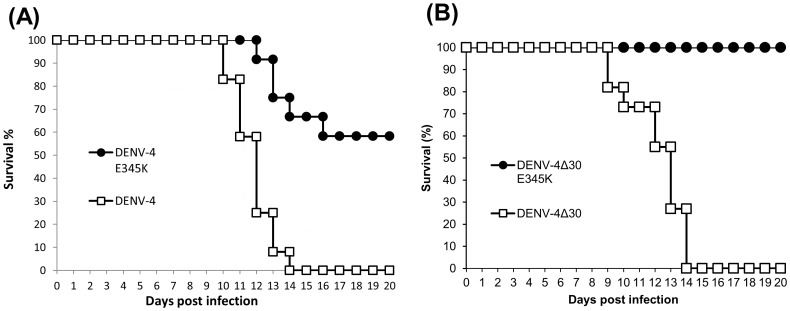
Results from neurovirulence assays of DENV-4 and DENV-4Δ30 mutant viruses in newborn ICR mice. Mice were intracranially inoculated with 10^4^ PFU or FFU of either virus. (A) DENV-4 E-E_345_K and DENV-4 wild type. (B) DENV-4Δ30 E-E_345_K and DENV-4Δ30 wild type viruses. Each treatment in this experiment was over 12 mice and in at least two litters. Each group consisted of at least 10 newborn mice per treatment. Bars represent means with standard deviation.

There is potential for virus genome changes from adaptive selection due to cell passaging, and mutations may affect cell tropism and virus virulence. We used newborn ICR mice to investigate neurovirulence in the E-E_345_K mutant derived from DENV-4 and DENV-4Δ30 infectious clones. The E-E_345_K mutant derived from the DENV-4 clone showed less virulence compared to its DENV-4 wild type. We observed a 58% survival rate for mice infected with the DENV-4 E-E_345_K mutant, compared to 0% for the DENV-4 (FIG. 5A). The E-E_345_K mutant derived from the DENV-4Δ30 clone had a complete loss in neuroviruelce compared to the wild type DENV-4Δ30 in newborn ICR mice (FIG. 5B). None of the mice infected with the DENV-4Δ30 E-E_345_K mutant died, compared to 0% survival rate infected with the DENV-4Δ30 (FIG. 5B).

### Rhesus monkeys viremia and anti-DENV neutralizing antibody titers

Since DENV-4 E-E_327_G mutant did not elicit either viremia or neutralizing antibodies in rhesus monkeys as reported previously [11], only DENV-4 wild type and DENV-4 E-E_345_K mutants were used to immunize six rhesus monkeys (three per group) with 10^5^ FFU dose. The results demonstrated that two out of three monkeys inoculated with DENV-4 E-E_345_K mutant had peak viremia during two to five days post inoculation, and the mean peak virus titer of these three monkeys was 17.7 FFU/ml with 2.3 days duration (Table 1). All of three monkeys inoculated with DENV-4 wild type elicited significantly higher viremia, ranging from 90.3 to 319.8 FFU/ml, with an average peak virus titer of 179.7 FFU/ml with 4.3 days duration (Table 1). Therefore, DENV-4 E-E_345_K mutant elicited almost 10-fold lower viremia in immunized monkeys. Anti-DENV neutralizing antibody titers in monkey serum samples at 14 and 28 days post inoculation were analyzed using PRNT assay. Our results indicated that the neutralization curves of monkeys immunized with DENV-4 wild type and DENV-4 E-E_345_K mutants elicited neutralizing antibodies by day 14 post-immunization, with titers ranging from 1∶44 to 1∶634 (Table 1). By day 28 post-immunization, five of six monkeys elicited similar levels of neutralizing antibodies with titers ranging from 1∶337 to 1∶679. One monkey immunized with E-E_345_K mutant elicited higher neutralizing antibodies (1∶1675) (Table 1).

**Table 1:** Rhesus monkeys immunized with DENV-4 wild type and DENV-4 E-E_345_K mutant viruses.

Group (FFU/monkey)	Monkey	Gender	Viremia at the indicated day postinoculation (PFU/ml)										Mean Peak titer (PFU/ml)	Mean Duration (d)	PRNT_50_ at the indicated day postinoculation		
			1	2	3	4	5	6	7	8	9	10			0	14	28
E345K (10^5^)	10R0463	M	0	8.4	0	0	0	0	0	0	0	0	17.7	2.33	0	43.7	1675.1
	11R0050	F	0	0	0	0	0	0	0	0	0	0			0	180.6	454.8
	10R0288	F	0	11.3	43.8	19	27	1	0	0	5.1	0			0	62.5	679.3
DENV4 (10^5^)	11R0052	F	319.8	225.6	121.2	37.5	0	0	0	0	0	0	179.7	4.67	0	634.3	489.1
	10R0479	M	90.3	30.2	66.2	38.2	62.8	55	0	0	0	0			0	230.7	337.1
	10R0512	F	0	113.3	47.1	129	55.3	0	0	0	0	0			0	347.8	488.4

### Protective immunity of Rhesus monkeys after challenge

To further confirm the protective immunity elicited by DENV-4 E-E_345_K immunization, the three immunized monkeys were challenged with 10^5^ DENV-4 wild type on 157 day post immunization. The results indicated that DENV-4 E-E_345_K did not elicit viremia in any of these three immunized monkeys (Table 2). The titers of anamnestic neutralizing antibody in sera collected at days 0, 15 and 30 post-challenge were analyzed. By day 15 post-challenge, three monkeys induced high-titer neutralizing antibodies ranging from 1∶702 to 1∶1884, and the neutralizing antibodies declined to the titers ranging from 1∶344 to 1∶866 at day 30 post-challenge (Table 2). Moreover, pre-challenge neutralizing antibodies titers show that all of the three monkeys had neutralizing antibodies ranging from 1∶ 380 to 1∶1428 at day 0 post-challenge (Table 2). All of the three monkeys immunized with DENV-4 E-E_345_K almost after 5 months post immunization still retained potent B cell memory responses, protected from live virus challenges, and induced high-titer anamnestic neutralizing antibodies.

**Table 2:** Protective immunity of rhesus monkeys immunized by E-E_345_K mutant virus*.

Group (FFU/monkey)	Monkey	Gender	Viremia at the indicated day postinoculation (PFU/ml)										Mean Peak titer (PFU/ml)	Mean Duration (d)	PRNT_50_ at the indicated day post challenge		
			1	2	3	4	5	6	7	8	9	10			0	15	30
E345K (10^5^)	10R0463	M	0	0	0	0	0	0	0	0	0	0	0	0	1428.2	1884.5	865.9
	11R0050	F	0	0	0	0	0	0	0	0	0	0			389.6	702.3	343.5
	10R0288	F	0	0	0	0	0	0	0	0	0	0			397.3	1296.1	424.6

*All animals were challenged with 10^5^ FFU of DENV-4 wild type on day 157 post-immunization.

## Discussion

The present study was based on Liu et al.'s (2008) report [9] that DENV-4 passaged in MRC-5 cells rapidly acquired an E-Glu_345_Lys (E-E_345_K) substitution in DIII of the E protein. We used site-directed mutagenesis to construct the E-E_345_K mutant viruses from DENV-4 and DENV-4Δ30 infectious clones and passaged in Vero or MRC-5 cells to demonstrate that genetic stability and replication kinetics were consistently presented in MRC-5 cells. The E-E_345_K mutant viruses showed greater attenuation in mouse neurovirulence, and were able to induce neutralizing antibodies in rhesus monkeys with an almost 10-fold lower titer of viremia as compared to the wild type virus. Monkeys immunized with the E-E_345_K mutant viruses were completely protected with no detectable viremia after live virus challenges with the wild type DENV-4.

According to sequencing analyses, E-E_345_K mutant viruses were stably maintained in MRC-5 cells but not in Vero cells. We also generated the E-E_327_G mutant viruses from both DENV-4 and DENV-4Δ30 infectious clones, propagated in MRC-5 cells, but appeared as a mix of G and A nucleotides at P4 sequences (data not shown). However, the E-E_327_G mutation was consistent in P0 and P4 Vero cells (data not shown). Additionally, we constructed the mutant viruses with other positively charged amino acid substitutions, E-Gly_345_Arg (E-E_345_R) and E-Gly_345_His (E-E_345_H), and the sequencing results indicated that only E-E_345_R but not E-E_345_H mutants were stably maintained in MRC-5 cells (data not shown). These findings suggest that a gain of more positive charges by lysine (K, pK of the side chain group is 10.5) and arginine (R, pKa of 12.5), but not histidine (H, pKa of 6.0), may account for DENV adaptation in MRC-5 cells.

Similar to other flaviviruses, DENV undergo adaptive mutations involving heparan sulfate binding for growth selection in FRhL, BHK and Vero cells.[11], [15], [16]. DENV-2 variants selected following passage in SW-13 or in BHK-21 cells contain a number of heparan sulfate-binding substitutions clustered in DII of the E protein [17]. The mutations E-K_291_ and E-K_295_ [18], E-E_345_K [9] and E-E_327_G [11] have all been identified as potential heparan sulfate-binding sites in DIII of the E protein. As the heparin inhibition on DENV-4 wild type, E-E_345_K and E-E_327_G mutant viruses may occur via electrostatic and hydrogen-bond interactions with the cationic amino acid side chains, we conducted a molecular structure modeling and surface mapping on E-DIII for E-E_345_K and E-E_327_G mutations according to the structural models determined by NMR spectroscopy (Protein Data Bank code 2H0P) [13] and cryo-electron microscopy (Protein Data Bank code 1THD) [11], [14]. The molecular modeling prediction of E-E_345_K and E-E_327_G mutations indicated that E-E_345_K and E-E_327_G mutations increase the net positive charge in adjacent areas (FIG. S2). Recombinant DIII proteins expressed from E.coli supported the findings that E-E_345_K and E-E_327_G mutations resulted in increased heparin bindings. The net positive charge increase is believed to accompany the creation of new binding sites for specific molecules as receptors for adsorption and infection.

Heparin and heparan sulfate are thought to be co-receptors for initial DENV adsorption and infection [19]. Recent studies demonstrated that DENV bound to negatively charged heparan sulfate proteoglycans of human umbilical vein endothelial cells [26], primary human dermal microvascular endothelial cells and HMEC-1 cells [27]. We found that heparin at the concentrations of 10 ug/ml inhibited the infectivity titers of DENV-4 wild type, E-E_345_K and E-E_327_G mutants, but not other types of GAGs such as hyaluronic acid, chondroitin sulfate A, chondroitin sulfate B and chondroitin sulfate C (FIG. 3). As heparin is composed of a repeating disaccharide subunit containing varying degrees of sulfation groups, we also examined four types of heparin disaccharide IV-A, I-H, III-S or I-S at three different concentrations (0.1 µg/ml, 1 µg/ml, and 10 µg/ml) mixed with DENV-4 wild types, E-E_345_K, and E-E_327_G mutants for the infectivity inhibition (FIG. S1). We did not observe significant levels of infectivity inhibition by the treatments with different forms of heparin disaccharides such as I-S, III-S, I-H, and IV-A at all three concentrations tested. These results suggest that the sufation pattern in the heparin disaccharides is unlikely related to the specificity of heparin inhibition on virus infectivity.

Knowledge of virulence and immunogenicity is important for the development of live-attenuated DENV vaccines. As rhesus monkeys are commonly used for studying DENV infections and vaccine development, we found that DENV-4 E-E_345_K mutant viruses elicited an almost 10-fold lower titer of viremia in rhesus monkeys as compared to DENV-4 wild type. The E-E_345_K mutant viruses were able to induce neutralizing antibodies in rhesus monkeys with an almost a 10-fold lower level of viremia as compared to the wild type virus. The discrepancy between mouse neurovirulence and monkey viremia induced by E-E_345_K and E-E_327_G mutant is unlikely associated with their enhanced heparin binding properties. Moreover, based on their elicited neutralizing antibodies in rhesus monkeys, the DENV-4 E-E_345_K mutant had a one-fold higher titer than DENV-4 wild type did. Comparing to other studies in rhesus monkeys, the neutralizing antibody titer elicited by E-E_345_K mutant was 5- to 7-fold higher than the chimeric DENV-2/Japanese encephalitis virus [31] and 2′-*O*-methyltransferase-defected DENV-2 [30], but 5- to 8-fold lower than the ChimeriVax-DENV-1 virus [28] and the DENV-1/2prM+E chimeric [29]. We observed that the monkeys immunized with DENV-4 E-E_345_K did not induce viremia post-challenge, and we found that the neutralizing antibodies elicited by E-E_345_K mutant after challenge was one-fold higher than 28 days post inoculation. However, we found that the neutralizing antibodies titer on days 30 post-challenge was lower than on days 15 post-challenge. Comparing to other studies, the neutralizing antibody titer elicited by E-E_345_K mutant after challenge was 2- to 4-fold higher than the chimeric DENV-2/Japanese encephalitis virus [31] and 2′-*O*-methyltransferase-defected DENV-2 [30], but 10- to 10-fold lower than the and the DENV-1/2prM+E chimeric [29]. The present studies also demonstrated that three monkeys immunized with E-E_345_K mutant had no detectable viremia after live virus challenges even after 5 months post immunization. The protection against live virus challenges also correlates with anamnestic neutralizing antibodies detected on days 15 and 30 post-challenges. Taken together, the present study demonstrated that the E-E_345_K mutation from MRC-5 cells can still induce potent immunogenicity in rhesus monkeys as compared to the complete loss of monkey immunogenicity by the E-E_327_G mutation reported from FRhL cell passage [11]. These results suggest that the DENV-4 E-E_345_K mutant propagated in MRC-5 cells may have potential for the use in live-attenuated DENV vaccine development.

## Supporting Information

Figure S1 **Effects of heparin and different form of heparin disaccharide on the inhibition of virus infectivity in Vero-E6 cells as determined by plaque percentages for (A) DENV-4 wild type, (B) DENV-4 E-E_345_K mutant and (C) DENV-4 E-E_327_G mutant.** Bars represent means with standard deviation from two independent sets of experiments. (* indicates statistical significance at p<0.05 in comparison to untreated controls).
(TIF)

Figure S2 **Molecular modeling and surface mapping of DENV-4 E protein electrostatic fields.** Blue and red denote positive and negative charges, respectively. White arrows: amino acid positions 345 and 327. Parental and variant structures were modeled into the DIII nuclear magnetic resonance-derived solution structure of DENV-4 E. Molecular modeling structure based on Protein Data Bank code 2H0P. Sum of partial charges analyses were carried using the PyMOL Molecular Graphics System software Version 1.1veal (Delano Scientific LLC).
(TIF)

Checklist S1 **ARRIVE Checklist.**
(PDF)
