# Supplementary figures and images for: Dengue Type Four Viruses with E-Glu345Lys Adaptive Mutation from MRC-5 Cells Induce Low Viremia but Elicit Potent Neutralizing Antibodies in Rhesus Monkeys
Source: PLoS One. 2014 Jun 24;9(6):e100130. doi: 10.1371/journal.pone.0100130 (PMC4069063; doi:10.1371/journal.pone.0100130)

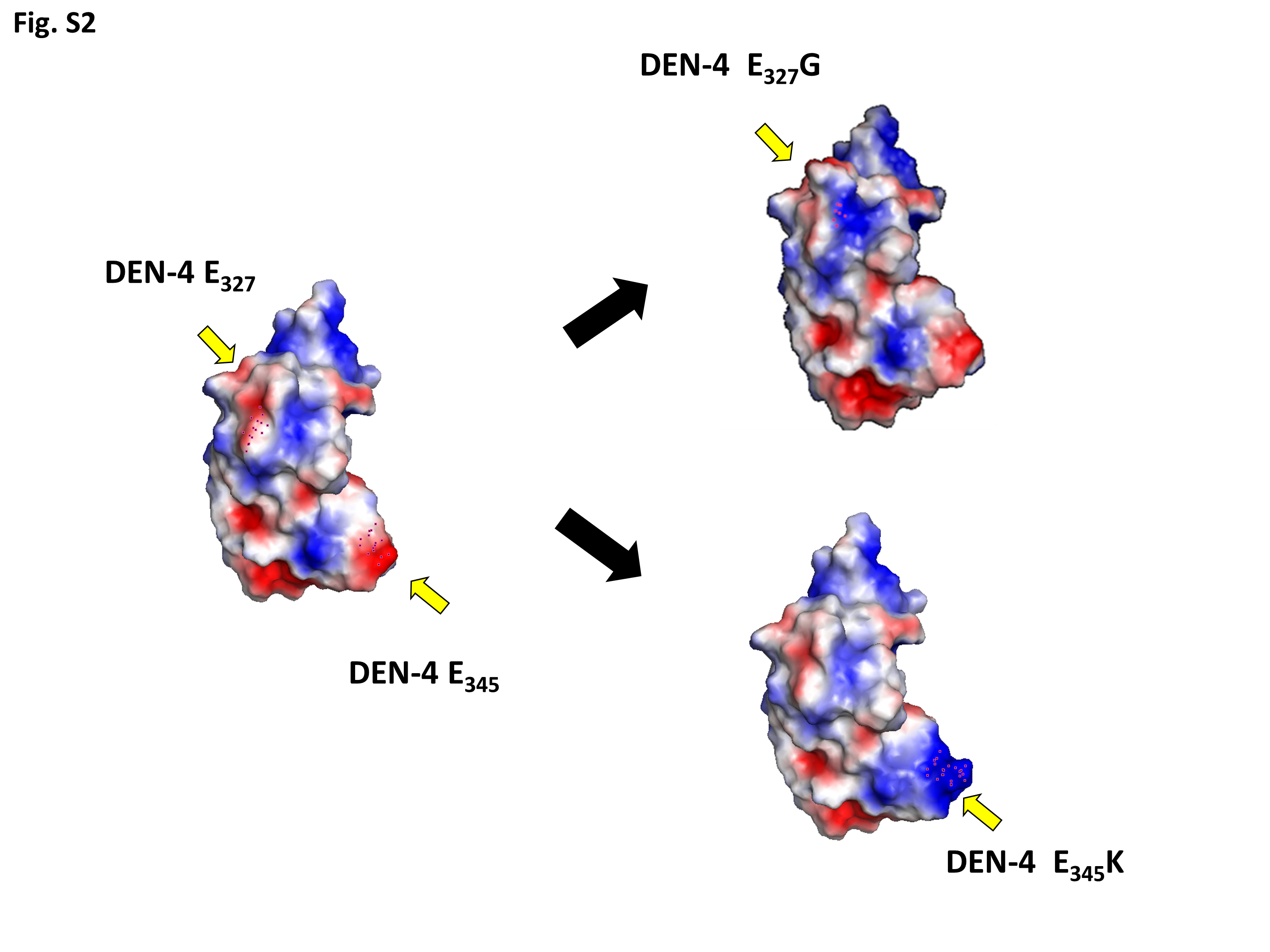

Supplement: Figure S2 — Molecular modeling and surface mapping of DENV-4 E protein electrostatic fields. Blue and red denote positive and negative charges, respectively. White arrows: amino acid positions 345 and 327. Parental and variant structures were modeled into the DIII nuclear magnetic resonance-derived solution structure of DENV-4 E. Molecular modeling structure based on Protein Data Bank code 2H0P. Sum of partial charges analyses were carried using the PyMOL Molecular Graphics System software Version 1.1veal (Delano Scientific LLC). (TIF) [file pone.0100130.s002.tif]
